# Leptin System in Obese Dog Skin: A Pilot Study

**DOI:** 10.3390/ani10122338

**Published:** 2020-12-09

**Authors:** Margherita Maranesi, Antonio Di Loria, Cecilia Dall’Aglio, Diego Piantedosi, Elvio Lepri, Paolo Ciaramella, Francesca Mercati

**Affiliations:** 1 Department of Veterinary Medicine, University of Perugia, via San Costanzo 4, 06126 Perugia, Italy; margherita.maranesi@unipg.it (M.M.); francesca.mercati@unipg.it (F.M.); 2 Department of Veterinary Medicine and Animal Productions, University Federico II of Napoli, via F. Delpino 1, 080137 Napoli, Italy; adiloria@unina.it (A.D.L.); diego.piantedosi@unina.it (D.P.); paociara@unina.it (P.C.)

**Keywords:** LEP, LEPR, RT-PCR, immunohistochemistry, integumentary system, dog

## Abstract

**Simple Summary:**

Obesity is a widespread phenomenon in pets and its growing trend is similar to the human one. It can be associated with skin pathologies but there is little information on this field in domestic animals. Since in obesity adipokine plasmatic levels changes, in this study leptin (LEP) system was evaluated in the skin of obese dogs to observe changes in peripheral tissue. LEP is a hormone produced mainly by the adipose tissue and its serum level may reflect body mass index and BCS. LEP is also expressed in the skin and it has a prominent role in the biology of this tissue promoting cell proliferation and regulating the wound healing process. Investigation performed in obese and normal-weight dogs evidenced LEP and leptin receptor (LEPR) immunostaining in several skin structures. As *LEP* expression regards, differences were non-significant, while the *LEPR* transcripts appeared 10 fold higher in obesedogs. No differences were observed in the composition of skin associated immune system. The obese group-increased *LEPR* expression suggests that the receptor modulates the system control. The LEP system changes in the skin under obesity conditions however, the exact role of LEP in obese dog skin needs further insights.

**Abstract:**

Obesity predisposes to several health problems including skin diseases. However, information on the relationship between obesity and skin disorders in pets is very scarce. Leptin (LEP) is mainly produced by adipose tissue and has a prominent role in skin biology. This study evaluated the LEP system in the skin of obese dogs compared to normal-weight animals. The investigation was carried out on 10 obese (Obese group) and 10 normal-weight (Normal-weight group) dogs through Real-time PCR and immunohistochemistry. Cells of skin associated immune system were also evaluated. No differences were evidenced between the two groups as well as skin inflammation. *LEP* differences were no significant, while *LEPR* transcript appeared 10-fold higher in obesedogs than in normal-weight ones. Immunostaining for both molecules was observed in several skin structures such as the epidermis, hair follicles, and glands. No differences appeared in the skin associated immune system composition. This study is a preliminary report showing that LEP system changes in obese dog skin. The increased *LEPR* expression observed in the obese group suggests that the receptor plays a modulating role in the system control. However, the exact role of LEPin the skin under obesity conditions needs further elucidation.

## 1. Introduction

Obesity is a chronic and multifactorial disease globally spread. In the obese, an accumulation of fat occurs which determines a strong excess of body mass such as to determine several chronic pathologies in humans [1] and animals [2]. Obesity represents an important health problem, which is accompanied by an increase in morbidity and mortality. Therefore, it is of particular interest to analyse and understand the pathophysiological role of adipose tissue in the development of diseases. Although concern with obesity has centred on humans, the disorder and its complications are also a growing problem in companion animals, particularly dogs [3,4]. As in humans, the main cause of weight gain is excessive food intake and poor physical activity [5]. Likewise, obesity has negative effects on dog health by causing a variety of diseases and a reduction in life expectancy [3,6]. Obesity-induced dogs showed similar features to human metabolic syndrome however, to distinguish the two syndromes, Tvarijonaviciute et al. [7] coined a new specific terminology for dogs, namely canine obesity-related metabolic dysfunction (ORMD). This is characterized by insulin resistance, altered lipid profiles, and mild hypertension, which are ameliorated by weight loss [8] as in other species. Canine ORMD is specifically related to the dog’s medical history and characteristic inflammatory and metabolic biomarkers modulations [7,9].

Indeed, obesity is considered a mild systemic inflammationand this has stimulated the study of possible correlations between pathology and the immune system [10]. In addition to acting as an energy reservoir, white adipose tissue is an endocrine organ capable of producing various types of hormones and molecules including inflammatory cytokines (TNF-alpha, IL-1B, IL-6, IL-8, IL-10, etc.) and proteins related to the immune function called adipokines [11]. These molecules seem implicated in the etiopathogenesis of some metabolic diseases such as type 2 diabetes, hypertension and cardiovascular diseases. Greater production of LEP and cytokines with proinflammatory action, such as interleukin 6 (IL-6) and “tumor necrosis factor-alpha” (TNF-α) occurs in obese [4,12].

Evidence suggests that LEP, a 16 kDa cytokine synthesized by adipocytes and by other non-adipose tissues [13], is a key hormone constituting the principal link between food intake and energy expenditure [14]. LEP exerts its effects on energy balance by acting on six different isoform receptors (LEPRa-f) localized in the brain, mainly the hypothalamus, muscle and other tissues [15], thus testifying its pleiotropic action [16]. Recently, LEPR has been identified in the skin of different species [17,18] including dogs [19]. LEP circulating levels are closely related to fat mass and LEP expression, and secretion is increased in obesity [13]. Blood LEP levels are proportional to insulin levels and inversely related to glucocorticoid concentration. Inflammatory cytokines including TNF, interleukin-1 and the inhibitory factor of leukemia (LIF) induce LEP secretion. LEP in turn plays a role in inflammatory processes: it activates monocytes and macrophages, enhances the production of pro-inflammatory cytokines (TNF-a, IL-6, IL-9) and promotes the differentiation of helper-1 T lymphocytes [16].

In the skin, LEP plays important roles both in physiological and pathological conditions [20]. It is involved in the renewal of the epidermis and hair follicles [21] and promotes wound healing by stimulating keratinocyte proliferation and angiogenesis [22]. Furthermore, LEP acts as a modulator of immune system activation [16]. The skin-associated immune system (SALT) is a complex network of cells belonging to both the innate and acquired immune system; keratinocytes, Langheran’s cells, dermal dendritic cells, mast cells and different subsets of lymphocytes (mainly T cells) are components of SALT. Obese subjects frequently show skin diseases. In obese men, LEP has been proposed as a pathogenetic cofactor of psoriasis as a stimulating factor for the local inflammatory process moreover its possible involvement in hypersensitivity reactions was suggested [23]. Obesity can also influence other skin-related disorders including ulceration, infection, poor wound healingandalso skin cancer [24,25]. Obese dogs show dermatological disorders starting from skin folds presence that predisposes to dermatitis [26] even if Lund et al. [27] did not observe a correlation between obesity and dermatopathies in dogs. Even if several functions of LEP in skin biology and in the pathogenesis of inflammatory skin diseases were hypothesized in humans and laboratory animals [28] its role in cutaneous pathophysiological processes remains unexplored in dogs. In this work, the expression of LEP and its receptor LEPR was analysed in dog skin, comparing obese and normal-weight subjects. Selected cells belonging to SALT were also evaluated. Furthermore, LEP serum levels as well as some haemato-chemical parameters were examined.

## 2. Materials and Methods

Ten obese and ten normal-weight, mixed breed dogs housed in a kennel in the Campania region (Italy),were referred to the Veterinary Teaching Hospital of the Department of Veterinary Medicine and Animal Productions of Naples for clinical evaluation and surgical routine procedures.

The body weight was evaluated (kg) for each dog. All animals, considered healthy on the basis of a complete clinical examination, complete blood count (CBC) and biochemical panel, were divided into two groups: obese dogs (Obese group; body condition score, BCS, ≥7/9) and normal-weight (Normal-weight group; BCS of 4–5/9) according to the nine-point body condition score (BCS) system [29]. Dogs were fed with a homemade, chicken-based high protein diet.

The study procedures were approved by the Ethical Animal Care and Use Committee (n.PG/2017/0099607) of the University of Naples Federico II.

### 2.1. Complete Blood Count (CBC) and Serum Biochemistry

A blood sample was obtained from each dog after jugular venipuncture using EDTA tubes and tubs with serum separator (Becton Dickinson, 1 Becton Drive, Franklin Lakes, NJ, USA). CBCs were performed using a semi-automatic cell counter (Genius S, SEAC Radom Group). After centrifugation at 327× *g* for 10 min, a semi-automatic chemical chemistry analyzer (OLOT, Spinreact, St. Esteve de Bas, Girona, Spain) was used to determine serum concentrations or activities of glucose, urea, creatinine, triglycerides, total cholesterol, alanine aminotransferase (ALT), alkaline phosphate (ALP), total bilirubin (T-Bil), gamma-glutamyl transferase (GGT), albumin and total serum proteins; serum proteins electrophoresis was also performed. Serum LEP concentrations were measured in duplicate using commercial canine ELISA kits (Millipore), following the manufacturer’s instructions.

### 2.2. Sample Collection

Skin specimens (c1 cm) were obtained from ventral abdominal region during neutering procedures under general anaesthesia, and were used to perform immunohistochemistry and Real-time PCR to evaluate both LEP and LEPR. Moreover, histochemical and immunohistochemical investigations were performed to evaluate leukocytes belonging to Skin Associated Lymphoid Tissue (SALT) namely B and T lymphocytes, macrophage-dendritic cells and mast cells.

Skin specimens were quickly fixed in 10% neutral buffered formalin solution in phosphate-buffered saline (PBS 0.1M, pH 7.4) and then processed for histological evaluation [30]. The fixed samples were dehydrated in graded ethanol, cleared in xylene and embedded in paraffin wax. Sections of 5 µm thick were cut and mounted onto poly-L-lysine coated glass slides and dried at 37 °C. Sections stained with Hematoxylin and Eosin were observed by light microscopy to exclude inflammatory or other skin lesions. GIEMSA stained slides were used to evaluate the number of mast cells.

For the molecular biology test, skin specimens were immediately frozen in liquid nitrogen and stored at −80 °C until it was time to measure the gene expression.

### 2.3. Immunohistochemistry

Immunohistochemistry was carried out as follows [19]: sections were rehydrated and dipped for 10 min in 3% H_2_O_2_ to reduce endogenous peroxidase activity. To perform antigen retrieval, sections were microwaved for 15 min in 10 mM citric acid (pH 6.0) or incubated with Historeveal (Abcam, Cambridge, UK) for 5 min as anti-LEPR antibody regards. The sections were then blocked with 1:10 normal serum (Table 1) for 30 min and incubated at room temperature with the primary antibody (Table 1) for 1h (for anti-CD20, Anti-CD3 and anti-Iba-1 antibodies) or overnight (for anti-LEP and anti-LEPR antibodies). After this primary step, the sections were incubated for 30 min with a biotin-conjugated secondary antibody (Table 1). The binding ofprimary antibodies wasvisualized using an avidin-biotin system (Vectastain ABC kit; Vector Laboratories, Burlingame, CA, USA) and diaminobenzidine (DAB) as chromogen (Vector Laboratories, Burlingame, CA, USA). All steps were performed at room temperature and the slides were incubated in a humid chamber. The sections were washed with PBS between all incubation steps, except after normal serum. Negative control sections were prepared by omitting the primary antibodies and incubating sections with normal rabbit or goat IgG (Novus Biological, Littleton, CO, USA). All sections were observed under a photomicroscope (Nikon Eclipse E800, Nikon Corp., Tokyo, Japan) connected to a digital camera (Nikon Dxm 1200 digital camera) and analysed by two independent investigators. The intensity of the LEP and LEPR staining of the skin reactive structures was graded in arbitrary units as follows: absent (0), moderate (1), strong (2) and very strong (3) [31]. The number of reactive cells to CD3, CD20 and Iba-1 were counted on 10 sequential highpower fields (400×) in superficial derma [32].

### 2.4. RNA Extraction and Real-Time PCR

Total RNA was extracted from the skin specimens of ten dogs from each experimental group as previously described [33]. Five µg of total RNA was reverse transcribed in 20 µL of iSCRIPT cDNA using random hexamer according to the protocol provided by the manufacturer (Bio-Rad Laboratories, Milan, Italy). Genomic DNA contamination was checked by developing a PCR without reverse transcriptase. Serial experiments were carried out to optimize the quantitative reaction, efficiency and Ct values. The optimal 25 µL PCR reaction volume contained 12.5 µL of iQ SYBR Green SuperMix (Bio-Rad Laboratories), 1 µL forward and reverse primers (stock concentration of 10 µM) and water to 25 µL. The primers used are listed in Table 2.

**Figure 1:**
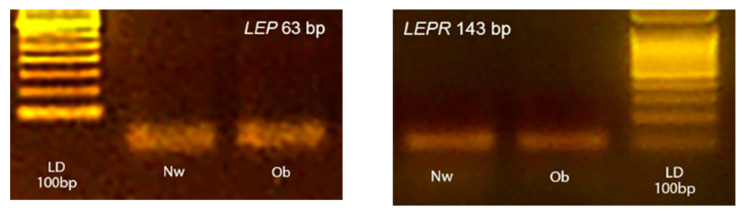
Representative photographs of typical 2% agarose ethidium bromide stained gels. The presence of the expected bp products yielded after RT-PCR using primers for target *LEP* and *LEPR* are showed. Lane LD is the kilobase DNA marker, lane Nw (Normal-weight) and Ob (Obese) identify two skin samples belonging to the two experimental groups.

All reagents were mixed as a master mix and distributed into a 96-well PCR plate before adding 2 µL of cDNA (10-fold diluted with water). For every PCR run, reaction controls without template and reverse transcriptase in RT were included as negative controls to ensure that RNA was free of genomic DNA contamination. The amplification fidelity of samples was also verified by agarose gel electrophoresis for two animals from each group (Figure 1). The images of gels were acquired by using a Kodak DC290 digital camera.

PCR was performed on an iCycleriQ (Bio-Rad Laboratories) with an initial incubation at 95C for 1.5 min, followed by 40 cycles at 95C for 15 s, 53C for 30 s, during which fluorescence data were collected. The threshold cycle (Ct value) was automatically computed for each trace. PCR products were purified and sequenced by Qiaquick PCR Purification Kit according to the manufacturer’s protocols (Quiagen Inc., Milan, Italy).

The beta-actin Ct housekeeping b gene (*BACT*) was determined in order to normalize sample variations in the amount of starting cDNA.

Standard curves were generated by plotting the threshold value (Ct) against the log Cdn a standard dilution (1/10 dilution) in nuclease-free water. The slope of these graphs was used to determine the reaction efficiency. Sample mRNA quantification was evaluated using iCycler system software, while mRNA gene expression was quantified using the 2^−ΔΔCT^ method [34,35]. The melting curve analysis was carried out, immediately after the PCR end cycle, to determine the specificity of each primer set. A melt–curve protocol was performed by repeating 80 heating cycles for 10 s, from 55C with 0.5C increments, during which fluorescence data were collected.

### 2.5. Statistical Analysis

Haemato-chemical datawere assessed by the Shapiro–Wilk test to evaluate for normality and a necessary logarithmic transformation was performed to compare the Obese and Normal-weight groups using Student’s *t*-test (SPSS version 20.0, IBM). *p*-Values < 0.05 were considered to be significant.

Data on gene expression and proteins were examined by ANOVA followed by Student–Newman–Keuls *t*-test. All values are means ± SD for each dog groups; differences were considered significant at *p* < 0.01 [36].

## 3. Results

The mean age, BW and BCS recorded in the Obese group (six males and four females) were 5.9 ± 1.2 years, 35.0 ± 9.2 kg and 7.9 ± 0.5, respectively; compared with 5.3 ± 1.3 years, 18.7 ± 3.4 kg and 5 ± 0, respectively for the Normal-weight group (six males and four females).

### 3.1. Hemato-Chemical Parameters

No significant differences for BW were noted with respect to sex. No significant differences in haematological parameters tested, which remained within physiological ranges, were observed between the two groups (Table 3).

In Obese group dogs, the biochemical panel evidenced a slight increase above the upper limit of the reference range of urea (n. 1), creatinine (n. 2), ALT (n. 1), ALP (n. 1), T-bil (n. 1), GGT (n. 4) and total serum proteins (n. 3). The results of serum biochemical profile are showed in Table 4; the two groups statistically differed in LEP and α-globulin fraction levels (*p* < 0.01), as well as GGT and T-Bil levels (*p* < 0.05).

### 3.2. Immunohistochemistry

**Figure 2:**
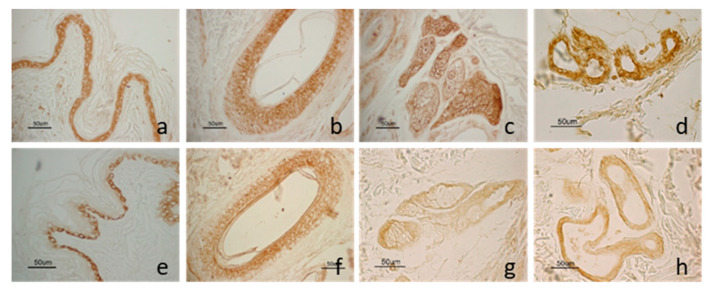
LEP and LEPR immunostaining in dog skin: LEP immunostaining in the epidermis (**a**); in the outer root cells of the hair follicle isthmic region (**b**); in the sweat gland (**c**) and the sebaceous gland (**d**). LEPR immunostaining in the basal cells of the epidermis (**e**); in the outer root cells of the hair follicle isthmic region (**f**); in the sweat (**g**) and the sebaceous gland (**h**).

LEP and LEPR staining was localized in some structures of the skin and showed a similar distribution pattern (Figure 2). The molecule and its receptor were observed in the epidermis, in the outer root cells of the hair follicles, in the sweat glands and the sebaceous glands. Moreover, LEP was also observed in vascular endothelial cells.

As epidermis regards, LEP staining was detected throughout all cell layers while LEPR immunoreactivity was present only in the basal layer. As hair follicle regards, staining was mainly localized in the isthmic region. Anagen hair follicles showed a wider percentage of positive cells compared with those in the regressive phase, both for LEP and LEPR. In the sweat gland, LEP staining was distributed widely on all gland cells while LEPR staining was mainly detected in the basal cells.

Immunostaining was not observed for LEP and LEPR in the control sections where the primary antibody was omitted or replaced with normal IgG.

Comparing the two analysed dog groups (Obese and Normal-weight), no significant difference (*p =* 0.67) was observed in the intensity of immunolabeling for LEP. Instead, as LEPR regards, immunopositivity appeared stronger in Obese dogs compared with Normal-weightdogs (*p <* 0.01) where immunostaining appeared rather weak. The results are summarized in Table 5.

Mast cell number, evaluated by Giemsa staining, ranged from 7–42 in the Obese group (mean 16 ± 12) and 10–85 (mean 30 ± 26) in the Normal-weight group. T lymphocytes immunoreactive to CD3 ranged from 20 to 72 (mean 43 ± 19) in the Obese group while from 10 to 115 (mean 37.7 ± 33) in the Normal-weight group. B lymphocytes immunoreactive to CD20 ranged from 0 to 4 (mean 0.7 ± 1.4) in the Obese group and 0–10 (mean 2.6 ± 3.4) in the Normal-weight group. Macrophages identified by Iba-1 immunoreactivity ranged from 18 to 128, (mean 60.8 ± 32) in the Obese group and 28–240 (mean 107.6 ± 42) in the Normal-weight group.

### 3.3. LEP and LEPR Gene Expression by Real-Time PCR

The *LEP* and *LEPR* transcripts were expressed in dog skin specimens independently of the group examined. There were no differences in *LEP* mRNA levels between the two groups, whereas the *LEPR* mRNA levels were 10-fold higher in the Obese group than in the Normal-weight one (Table 6).

## 4. Discussion

The principal aim of this study was to evaluate LEPand LEPR presence and localization in the skin of Obese dogs and verify, whether their expression is modulated with different patterns with respect to Normal-weight dogs. This study, which stems from our previous one on LEPR in the epidermis and skin appendages in dogs [19], is the first to investigate the functional expression of LEPand LEPR in the skin of obese dogs correlating these findings to LEP circulating levels.

Obese dogs evaluated showed a marked increase inthe LEP circulating levels compared to Normal-weight animals (*p* < 0.01). The development of hyperleptinemia and LEP resistance has been well documented in humans and domestic animals in overweight and obesity conditions [39]. In addition to the role in the regulation of energy metabolism, LEP has a pivotal role in the regulation of the immune response, neuroendocrine mechanisms and hematopoiesis [40]. Serum LEP concentration is considered a reliable marker of adiposity in dogs regardless of age, gender and breed variations, and decreases with weight loss [41,42], and thereby it could be a potentially useful tool to assess the obesity status also in the clinical setting [9].

Regarding the metabolic status, Obese dogs showed increased weight, elevated serum α-globulin fraction and had higher serum LEP concentrations than Normal-weightdogs (*p* < 0.01). The elevated serum α-globulin fraction, detected in the Obese group, could be due to an increase in specific acute phase proteins, indicative of a potential inflammatory state previously described both in obese human and dog patients [43,44]. The observed increase in GGT and T-Bil could be related to cholestasis and/or biliary retention, common laboratory findings reported in obese dogs [45,46]. There is evidence that obesity in humans leads to the development of biliary diseases, because of excessive hepatic secretion of cholesterol, subsequent supersaturation of bile, an increase in gallbladder volume, and impairment in gallbladder contraction [47]. No increase in cholesterol and triglycerides was detected in the Obese group, probably due to the chicken protein-based diet administrated to all dogs enrolled. In humans, the high-protein diet commonly used for weight loss is able to control serum triglyceride levels and cholesterol, although lipid disorder may depend not only on the amount of carbohydrate restriction but also on the control of the intake of saturated fat [48]. Moreover, it is noteworthy to underline that only a subset of obese dogs falls within the ORMD, for which hyperlipidemia represents one of the main characters, and in the same way in obese people is known a condition defined as “metabolically healthy obesity” [49].

At the skin level, LEP and LEPR immunostaining were observed in several structures including the epidermis, hair follicles, sweat glands, sebaceous glands and endothelial cells. Obtained results, referring to molecule localization, are comparable and confirm features previously described for both the ligand [28] and the receptor [19]. Through Real-Time PCR, no difference in *LEP* expression was evidenced between the two groups. Instead, a great difference was observed for the receptor that appeared ten times higher (*p* = 6.18 × 10^−6^) in Obese animals. In agreement with gene expression analysis, immunolabeling data showed no significant difference for LEP protein while the receptor staining appeared weak in Normal-weight dogs and strong (*p* < 0.01) in Obese dogs. Carmina et al. [50] observed that LEP expression decreases with increasing body mass index both in the omentum and in the subcutaneous fat, while Viesti et al. [51] did not detect a significant difference in LEP expression in the omentum and liver of the obese subjects compared tocontrol.Obesity is related to several skin diseases in the human species including poor wound healing and increased risk of dermatitis, such as psoriasis [25]. LEP promotes wound healing but LEP resistance, associated with obesity, may contribute to the pathophysiology of impaired wound repair [25]. However, the higher *LEPR* expression evidenced in obese dogs would suggest a better response of skin to serum LEP. Accordingly, Sabol et al. [52] demonstrated that postnatal overfeeding and obesity were associated with improved wound healing in adolescent male rats.

LEP has a role in acute and chronic inflammation via the regulation of cytokine expression [16,53] and it contributes to the proinflammatory environment observed in obesity and psoriasis in humans [54]. However, a low-grade chronic inflammatory state determined by measuring circulating TNF-α and IL-6 was not detected in obese dogs [16]. In dog skin, no differences were observed in the number of the cells of the SALT between Obese and Normal-weight groups. Nevertheless, it must be observed that the number of Iba-1 positive cells, likely represented by dendritic interstitial cells [55], was lower in Obese dogs than in Normal-weight ones; this result could be an incidental finding related to external influences or could reflect a lower activity of natural immune system in the skin in obese dogs; finally, it may be positively related to the reduced, even not significant, *LEP* expression observed, in the present study, at the skin level. It is widely explored that LEP acts with several mechanisms on adaptive immunity, and particularly on different T cell populations [39]. Palatucci et al. [56] reported that obese Labrador Retrievers are characterized by the inverse correlation between LEP serum concentration and circulating Treg levels, a specific immune regulatory T cell population. Reduced Treg cells were observed in visceral adipose tissue of obese mice and humans [39]. In this context, further studies are necessary to investigate whether there is a difference in the regulatory role of LEP on the immunological network at the level of the various peripheral sites, such as the skin.

Obese dogs showed an intense LEP and LEPR immunostaining at the level of sweat and sebaceous glands. *LEP* and *LEPR* expression were already described in these structures [19,57] that were both proposed as a source and target of LEP. Human obese patients sweatmore profusely and an increase in sweat gland activity was suggested [58]. In dogs, apocrine sweat glands are associated with hair follicles and their secretion is poured on the skin surface together with sebum as an antimicrobial emulsion. Sweat glands are also involved inpheromones and chemical signals production aimed at social communication [59]; these functions could undergo modifications in obesity conditions. LEP modulates the development of sebaceous glands as well as sebum production [57]. An altered amount and composition of sebum are associated with skin diseases such as acne vulgaris and atopic dermatitis in humans. Endocrine diseases in the dog commonly manifest with dermatological lesions including seborrhea [60]. The stronger expression of *LEPR* in obese skin dogs may suggest a higher sensibility of both skin glands to the action of circulating LEP levels.

It can be supposed that the LEP receptor changes its expression following the stimulation of the increased plasma LEP since hormone local production was not changed. The increased *LEPR* expression observed in the Obese group suggests that the receptor plays a modulating role in the skin system control and that serum LEP may have a role in the pathogenesis of skin disease associated with obesity [61]. However, Arnold et al. [62] have shown that among the different isoforms of LEPR (LEPRa, b, c, d, f: transmembrane), there is also a soluble form of the LEP receptor (LEPRe) capable of modulating the bio-availability and the consequent action of LEP. This is an interesting issue to be further considered. The presence of a soluble receptor form, which captures the available plasma LEP, could explain the general impaired full functionality of LEP (LEP resistance), as well as, the skin reaction represented by LEPR over-expression, thus testifying to a possible involved cause in the well-documented skin disorders in obese subjects [61].

There are no reports of LEP system expression in the skin of obese subjects and few reports on differences at peripheral sites. LEP downregulation of LEPR expression was suggested as one of the LEP resistant mechanisms for maintaining obesity [63]. Despite this, no change in Ob-Rb gene expression was found in mice on a high-fat diet [64]; Münzberget al. [65], even when shown to be fully LEP resistant [66]. Priego et al. [67] described a reduced or increased variation of LEPR according to sex and different depots of white adipose tissue on rats. Moreover, significant differences of LEPR gene expression were not evidenced in the hypothalamus [68], in subcutaneous fat, liver and visceral fat [51]. Literature data suggest that possible differences in *LEP* and *LEPR* expression in peripheral tissues, as well as species differences in obesity condition, should be taken into consideration. Surely, the high expression of the LEPR observed in this study attests that the skin is a sensitive tissue to changes in the LEP system that occurs in obese dogs. Further research we need to better understand this mechanism.

## 5. Conclusions

The skin is not only a barrier between the inner and external environment but also a hormonally and metabolically active organ characterized by its own activity and secretion. Skin physiological balance can be affected by various external and internal factors including obesity. Skin is also a significant source of pro- and anti-inflammatory factors associated with the course of the inflammatory process, i.e., adipocytokines. The numerous and complex functions of these molecules in the organism results in linkages between obesity and metabolic disorders.

Obesity and its associated diseases are a growing concern in pets even if there are a few reports that investigated this relation in the skin. This work shows that the LEP system changes in the dog skin under the obesity condition. In particular, the skin increases its sensitivity to the LEP by a modulating role of the LEPR. This preliminary study opens interesting questions on the modification and action of adipokines in the skin in obesity conditions. However, the exact role of LEP in the skin of obese dogs needs further elucidation.

## Figures and Tables

**Table 1:** Source and working dilutions of the antisera used.

Antisera	Working Dilutions	Sources
normal goat serum	1:10	S-1000, Vector Laboratories, Burlingame, CA, USA
normal horse serum	1:10	S-2000, Vector Laboratories, Burlingame, CA, USA
polyclonal rabbit anti-LEP	1:100	abx177326, Abbexa Ltd., Cambridge, UK
polyclonal goat anti LEPR	1:200	ab 50424, Abcam, Cambridge, UK
polyclonal rabbit anti-CD20	1:200	RB9013-P; ThermoScientific, Waltham, MA, USA
polyclonal rabbit Anti-CD3	1:200	A0452; DakoCytomation, Carpentera, CA, USA
monoclonal mouse anti-Iba-1	1:50	MABN92; Merck Millipore, Damstadt, Germany
horse anti rabbit IgG biotin conjugated	1:200	BA-1100; Vector Laboratories, Burlingame, CA, USA
horse anti-goat IgG biotin conjugated	1:200	BA-9500; Vector Laboratories, Burlingame, CA, USA
goat anti-mouse IgG biotin conjugated	1:200	Ab6788 Abcam, Cambridge, UK

**Table 2:** Primers for leptin (*LEP*), leptin receptor (*LEPR*) and beta-actin Ct (*BACT*) housekeeping gene used for Real-Time PCR quantification.

Gene	NCBI seq, ref.		Primers
*LEP*	NM_001003070.1	F	ACCGTATGGGTGTCCTTTATCCT
		R	AGAGTGGCTCTGTGGTGTGAGA
*LEPR* [19]	NM_001024634.1	F	CTTTTGCCTGCTGGAATCTC
		R	TTGCTCCAAAAGCAACAGTG
*BACT*	NM_001195845.2	F	CTTCCAGCCTTCCTTCCTGG
		R	CCAGGGTACATGGTGGTTCC

**Table 3:** Haematological results in Obese and Normal-weight dogs.

Parameter	Unit	Reference Ranges	Ob Group	Nw Group	*p*
RBCs	(×10^6^/µL)	5.5–8.5	7.7 ± 0.57	7.6 ± 0.36	0.255
HGB	g/dL	12–18	16.8 ± 0.63	17.2 ± 0.49	0.061
HCT	%	37–55	52.1 ± 1.94	52.0 ± 2.08	0.456
MCV	fL	60–77	67.9 ± 6.92	68.8 ± 4.74	0.370
MCH	pg	20.5–24.2	21.8 ± 2.30	22.7 ± 1.36	0.162
MCHC	%	32–36	32.1 ± 1.25	33.0 ± 1.85	0.113
WBCs	(×10^3^/µL)	6–17	10.7 ± 2.63	11.8 ± 1.70	0.151
PLTs	(×10^3^/µL)	200–500	354.3 ± 78.96	311.8 ± 70.60	0.110

Data are expressed as mean ± standard deviation. RBCs: Red blood cells; HGB: Haemoglobin; HCT: alanine aminotransferase; MCV: Mean Corpuscular Volume; MCH: Mean Corpuscular Haemoglobin; MCHC: Mean Corpuscular Haemoglobin Concentration; WBCs: White blood cells, PLTs: platelets. Ob: Obese dog group; Nw: Normal-weight dog group. Reference ranges: Rizzi et al. [37].

**Table 4:** Serum biochemical analysis and serum LEP profile in Obese and Normal-weight dogs.

Parameter	Unit	Reference Ranges	Ob Group	Nw Group	*p*
Glucose	mg/dL	65–118	82.6 ± 8.41	77.3 ± 10.14	0.110
Urea	mg/dL	21–59	41.5 ± 11.15	37.7 ± 7.56	0.192
Creatinine	mg/dL	0.5–1.5	1.4 ± 0.2	1.3 ± 0.14	0.067
T-Chol	mg/dL	135–270	151.2 ± 34.84	154.5 ± 30	0.411
TG	mg/dL	20–112	48.4 ± 15.99	38.9 ± 4.79	0.052
ALT	U/IL	21–102	39.3 ± 24.34	37.2 ± 10.72	0.403
GGT	UI/L	1.2–6.4	5.1 ± 2.85	3 ± 1.05	0.025 *
ALP	UI/L	20–156	70.6 ± 49.15	66.7 ± 35.1	0.420
T-Bil	mg/dL	0.1–0.5	0.3 ± 0.19	0.1 ± 0.02	0.016 *
TP	g/dL	5.4–7.1	7.1 ± 0.62	6.8 ± 0.42	0.144
Alb	g/dL	2.6–3.3	3.5 ± 0.27	3.4 ± 0.40	0.163
α1-glob	g/dL	0.2–0.5	0.2 ± 0.05	0.2 ± 0.02	0.004 **
α2-glob	g/dL	0.3–1.1	1.0 ± 0.13	0.9 ± 0.15	0.087
β1-glob	g/dL	0.7–1.3	0.8 ± 0.23	0.9 ± 0.35	0.135
β2-glob	g/dL	0.6–1.4	0.8 ± 0.15	0.8 ± 0.23	0.323
γ-glob	g/dL	0.5–1.3	0.7 ± 0.25	0.6 ± 0.14	0.054
LEP	ng/mL	-	14.6 ± 5.88	6.3 ± 4.07	0.003 **

Data are expressed as mean ± standard deviation. T-Chol: total cholesterol; TG: total triglycerides; ALT: alanine aminotransferase; GGT: gamma-glutamyl transferase; ALP: alkaline phosphate; T-Bil: total bilirubin; TP: total proteins, Alb: albumins; α1-glob: α1-globulins;α2-glob: α2-globulins; β1-glob: β1-globulins; β2-glob: β2-globulins; γ-glob: γ-globulins; LEP: leptin. Ob: Obese dog group; Nw: Normal-weight dog group. Reference ranges:Kaneko et al. [38]. * *p* < 0.05; ** *p* < 0.01.

**Table 5:** Intensity of LEP and LEPR immunostaining in Obese and Normal-weight dog skin sections.

	LEP		LEPR	
Groups	Mean	SD	Mean	SD
Ob	1.91	0.76	1.82	0.66
Nw	1.72	1.04	0.66	0.42
*p* * Ob vs. Nw	0.67		0.0016	

The intensity of staining in tissue sections was sorted on a scale ranging from 0 (negative) to 3 (very strong). The means ± SD of LEP and LEPR protein immunostaining intensity were calculated for 10 animals/group. * Significantly different values were considered at *p* < 0.01 between Obese and Normal-weight groups. LEP: leptin; LEPR: leptin receptor; Ob: Obese dog group; Nw: Normal-weight dog group.

**Table 6:** Mean mRNA levels and standard deviation values (SD) of *LEP* and its receptor in Obese and Normal-weightskin dog.

	LEP		LEPR	
Groups	Mean	SD	Mean	SD
Ob	4.70	5.10	5.15	0.95
Nw	9.06	7.01	0.47	0.32
*p* * Ob vs. Nw	0.37		6.18 × 10^−6^	

Real-time PCR mRNA expressions for *LEP* and *LEPR* in skin collected in Obese and Normal-weight dogs. Data are represented as the fold changes of mRNA expression in Obese dogs to those in Normal-weight dogs to a*beta-actin* housekeeping gene. The relative abundances of target genes were calculated using the 2^−ΔΔCT^ method. The means ± SD of *LEP* and *LEPR* mRNA expression levels for the three mRNA measurements were calculated for 10 animals/group. * Significantly different values were consideredat *p* < 0.01 between Obeseand Normal-weight groups. *LEP*: leptin; *LEPR*: leptin receptor; Ob: Obese group; Nw: Normal-weight group.
